# How to Effectively Reduce Honey Adulteration in China: An Analysis Based on Evolutionary Game Theory

**DOI:** 10.3390/foods12071538

**Published:** 2023-04-05

**Authors:** Xiao-Wei Zhang, Letian Xu, Si-Yi Wang, Lin Wang, Derek W. Dunn, Xiaoping Yu, Xinping Ye

**Affiliations:** 1 College of Life Sciences, Shaanxi Normal University, Xi’an 710119, China; 2 State Key Laboratory of Biocatalysis and Enzyme Engineering, School of Life Sciences, Hubei University, Wuhan 430062, China; 3 School of Modern Posts, Xi’an University of Posts & Telecommunications, Xi’an 710061, China; 4 Ministry of Education’s Key Laboratory of Poyang Lake Wetland and Watershed Research, School of Geography and Environment, Jiangxi Normal University, Nanchang 330022, China; 5 College of Life Sciences, Northwest University, Xi’an 710069, China

**Keywords:** evolutionary game theory, apiculture, bee product supervision, adulteration, food security

## Abstract

Apiculture has been greatly developed in recent years in China. Beekeeping cooperatives and honey manufacturing enterprises have increased rapidly. As a result, a variety of honey products have entered the market, adding vitality to the food economy; however, the adulteration of honey products is on the rise in China. Previous attempts to control the adulteration of honey products mostly relied on technical, product-specific measures, and there was a lack of modeling research to guide the supervision of the honey product industry. In order to help local governments to better control the adulteration of honey products from a management perspective, this paper establishes an evolutionary game model composed of beekeeping cooperatives, honey product enterprises, and local governments. Through stability analysis and model simulation, we found that local government subsidies to cooperatives have little impact on the game system. Local government penalties to cooperatives and price adjustments of unadulterated raw honey by cooperatives are effective management tools to reduce the adulteration behavior of cooperatives. Local government penalties for enterprises are an effective management tool to reduce the adulteration behavior of enterprises. This research provides useful information for government agencies to design appropriate policies/business modes so as to promote sustainability and the healthy development of the honey product industry in China.

## 1. Introduction

As an important part of modern agriculture, apiculture is a clean method of production that provides large amounts of nutrient-rich honey products with high economic value for society. Honey products have become indispensable food resources in people’s daily lives. China is the largest beekeeping country in the world, and there are more than 300,000 farmers engaging in beekeeping [1]. The bee farmers in China have raised more than 9 million colonies of *Apis mellifera* [1]. In addition to *Apis mellifera*, the breeding scale and honey production of the native honeybee *Apis cerana* are also very impressive. According to the literature, the number of *Apis cerana* raised in China accounts for about 1/3 of the total number of honeybee colonies [2]. In China’s remote agricultural areas, the development of apiculture plays an increasingly important role in reducing natural environmental damage and improving the living conditions of farmers [3]. In areas where bee farmers are concentrated, the local governments help them to set up beekeeping cooperatives. The establishment of beekeeping cooperatives promotes the sale of raw honey to enterprises and prevents the disadvantage of individual farmers in trading with companies. Li et al. sampled and analyzed 535 beekeeping cooperatives in China, 180 of which had between 1000 and 5000 bee colonies and 40 of which had over 10,000 bee colonies. A total of 95.7% of beekeeping cooperatives were registered, and 78.31% of beekeeping cooperatives were established after 2011 [4]. Li’s results show that beekeeping cooperatives in China are becoming increasingly standardized and have also become the main mode of raw honey production.

With the rapid development of apiculture in China, the quantity of raw honey produced by beekeepers has greatly increased, and many honey product manufacturing enterprises have mushroomed. China’s honey products not only meet domestic demand but are also exported to many countries overseas, and overall production is among the largest in the world. In 2019, the export volume of honey products was approximately 124,494 tons, and their export value was USD 294 million [5]. Due to the popularity of honey products and the increasing demand by urban residents, interests drive adulterated products to constantly emerge in the market.

There are various types of bee products, such as honey, royal jelly, bee pollen, propolis, wax, bee venom, and bee bread [6]. The most common and most consumed bee products in the market are honey products. Studies have proven that honey has anti-inflammatory, antibacterial, and antioxidant properties and that it helps lower blood pressure and blood lipids. Therefore, honey products are also widely used as ingredients in apitherapy and healthcare food [7,8,9]. There are many types of honey products; they vary depending on nectar sources and processing techniques. There are adulterated versions of every type of honey product, which are linked to the production and sales of unadulterated honey products. Furthermore, beekeeping cooperatives and honey product enterprises usually vary greatly in size, and even the same types of products lack unified industrial standards. As a result, adulteration may occur in all aspects of honey production. As for raw honey, the most common adulteration practices are feeding bees sugar and adding sweeteners (such as caramel, fructose, and corn syrup) to honey [10]. For honey enterprises, there are many ways to produce adulterated honey products, including the use of adulterated raw honey as raw materials, the blending of syrups into products, and the synthesis of chemical materials. [6] Moreover, the government’s supervision of honey products in China is still in its infancy. The above factors provide fertile ground for the adulteration of raw honey and honey products, and it is often difficult for ordinary consumers to identify authentic honey products, which increases the prevalence of adulterated honey products. The spread of adulterated honey products in China has led to a distrust of honey products and doubts about the credibility of the government. According to the official report of the European Union, one coordinated action confirmed that a significant part of honey imported into the EU is suspicious of adulterated products (46% based on 320 samples), and the highest absolute number of suspicious consignments originated from China (74%). [11] Products exported overseas are frequently returned due to their substandard quality, which not only causes economic losses but also has a negative impact on the international reputation of manufacturing in China.

In order to tackle the issue of adulterated honey products, market regulators and third-party testing agencies have developed a series of authenticity testing methods for honey products, including sensory identification [12,13,14], DNA-based approaches [15,16,17], isotope-ratio mass spectrometry (IRMS) [18,19,20,21], nuclear magnetic resonance (NMR) spectroscopy [22,23,24], near-infrared (NIR) spectroscopy [25,26,27,28] and mass spectrometry (MS) [29,30,31]. However, these measures are usually only used in specific adulteration cases. Reducing the adulteration of honey products depends on the supervision and regulation of the whole industry by local governments, and only regulation by local governments may possibly eliminate the prevalence of adulteration in the honey product industry.

The evolutionary game model has been widely used in drug supervision and management [32], public transportation management [33], new energy use promotion [34], the management of the utilization of wild animal and plant resources [35], and other fields [36]. The evolutionary game model has positive guiding values for practical management by simulating and predicting the behavior of different stakeholders. The theoretical model predicts that the players of the game will gradually reach equilibrium, but changing the conditions will speed up or slow down the evolution time for different stakeholders to reach equilibrium [37]. Therefore, changing the external conditions can provide guiding significance to all parties involved. Different from the supervision of drugs and public transportation, the supervision of honey products (SHP) involves the entire industrial chain, from raw honey to honey products; therefore, honey product supervision is more complicated. This paper established an evolutionary game model of different stakeholders in the regulation of honey products in China (SHP-game) and analyzed the influence of different factors on the evolution process of the game system to provide guidance for the regulation of honey products in China and to accelerate the reduction of adulteration in the market from the perspective of management.

## 2. Evolutionary Game Model

### 2.1. Assumptions of Game Model

(1) Problem description

**Figure 1:**
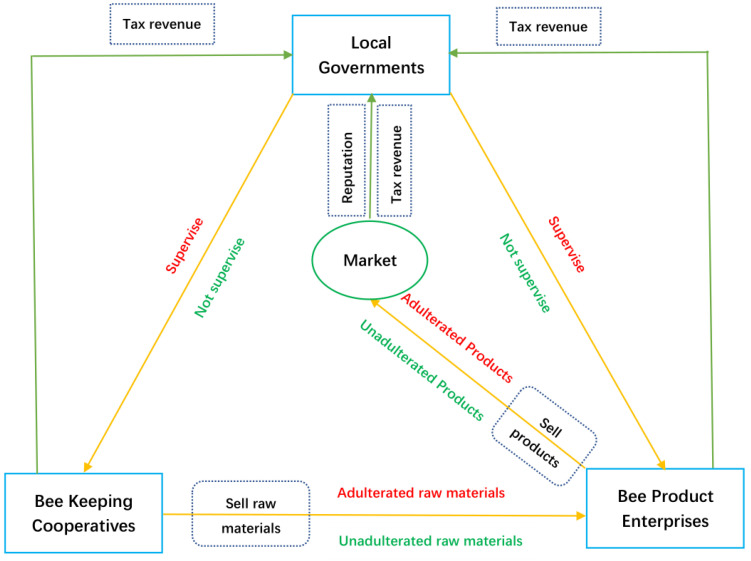
The relationships between different stakeholders in the SHP-game model.

The relationship between different stakeholders in the SHP-game model is shown in Figure 1.

There are three main participants in the SHP-game model, namely beekeeping cooperatives (BCs), honey product enterprises (HEs), and local governments (LGs). BCs provide raw honey to BEs. BEs produce various commercial honey products and put them on the market, while the LGs supervise the behaviors of BCs and HEs (Figure 1). In China, BCs are established by bee farmers through certain agreements. In many areas, raw honey materials are uniformly sold by BCs to HEs. In this way, bee farmers can guarantee the sales of raw honey and avoid losses caused by price fluctuations; at the same time, HEs can guarantee a sufficient supply of raw honey. In order to encourage BCs to supply unadulterated raw honey to BEs and to encourage HEs to produce products that meet quality standards, the LGs have corresponding subsidies for BCs and HEs. In contrast, LGs punish the BCs and HEs that adulterate honey to stop this practice. The parameters of relevant stakeholders in the SHP-game model are shown in Table 1.

(2) Model hypothesis

**Figure 2:**
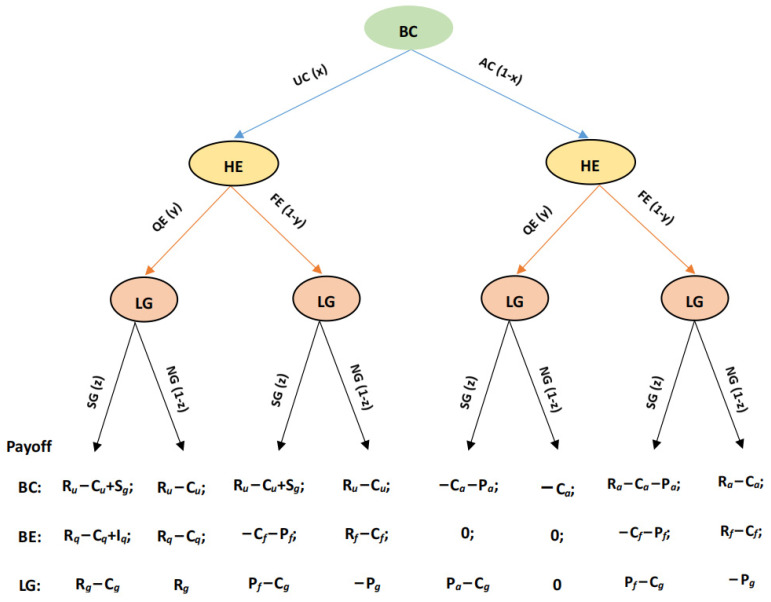
Game tree and payoff matrix of BCs, HEs, and LGs.

Based on the above relationship, some complex conditions can be simplified without changing the nature of the problem, and the following assumptions are made:

① The three parties are all participants of bounded rationality, and the strategy selection gradually evolves to the optimal strategy over time; 

② The strategies set for BCs are to produce unadulterated raw honey (UC) and produce adulterated raw honey (AC); the strategy set for HE is to produce qualified bee products (QE) and produce fake (adulterated) bee products (FE); the strategy set for LG is to supervise (SG) and not supervise (NG). The game tree and payoff matrix are shown in Figure 2;

③ The cost of producing unadulterated honey (C_u_) is greater than that of adulterated materials (C_a_), and the price of unadulterated honey (R_u_) is higher than that of adulterated materials (R_a_);

④ The cost of producing qualified products (C_q_) is greater than that of adulterated products (C_f_), and the price of qualified products (R_q_) is higher than that of adulterated products (R_f_);

⑤ The cooperatives, enterprises, and local governments act to maximize their interests.

### 2.2. Replicator Dynamic Equation

Evolutionary game theory is a combination of game theory and dynamic evolutionary process analysis, with an emphasis on dynamic equilibrium [37]. According to evolutionary game theory, if the payoff of a certain strategy is higher than the average payoff of the population, the percentage of individuals adopting this strategy in the population will gradually increase, and its growth rate can be obtained by the replicator dynamic differential equation. Thus, the replicator dynamic equation describes the variation in the frequency of a particular strategy adopted by a population over time [38]. The higher the replicator dynamic value, the more the proportion of the strategy will increase.

According to the above payment matrix, the expected payoff of BCs that use the UC strategy (E_11_), AC strategy (E_12_), and average expected payoff of BCs (E_1_) can be calculated, respectively, by the following:

(1) $$E_{11}=\mathrm{yz}(R_{u}-C_{u}+S_{g})+y\left(1-z\right)\left(R_{u}-C_{u}\right)+\left(1-y\right)z\left(R_{u}-C_{u}+S_{g}\right)+\left(1-y\right)\left(1-z\right)\left(R_{u}-C_{u}\right)$$

(2) $$E_{12}=\mathrm{yz}(-C_{a}-P_{a})+y\left(1-z\right)\left(-C_{a}\right)+\left(1-y\right)z\left(R_{a}-C_{a}-P_{a}\right)+\left(1-y\right)\left(1-z\right)\left(R_{a}-C_{a}\right)$$

(3) $$E_{1}={\mathrm{xE}}_{11}+\left(1-x\right)E_{12}$$

The replicator dynamic equation of UC strategy is

(4) $$f\left(x\right)=dx/dt=x\left(E_{11}-E_{1}\right)=x\left(1-x\right)\left(E_{11}-E_{12}\right)=x\left(1-x\right)\left[{\mathrm{zS}}_{g}+{\mathrm{zP}}_{a}+yR_{a}+R_{u}-C_{u}+C_{a}-R_{a}\right]$$

The expected payoff of HEs that use the QE strategy (E_21_), FE strategy (E_22_), and average expected payoff of HEs (E_2_) can be calculated, respectively, by the following:

(5) $$E_{21}=\mathrm{xz}(R_{q}-C_{q}+I_{q})+x\left(1-z\right)\left(R_{q}-C_{q}\right)$$

(6) $$E_{22}=xz\left(-C_{f}-P_{f}\right)+x\left(1-z\right)\left(R_{f}-C_{f}\right)+\left(1-x\right)z\left(-C_{f}-P_{f}\right)+\left(1-x\right)\left(1-z\right)\left(R_{f}-C_{f}\right)$$

(7) $$E_{2}={\mathrm{yE}}_{21}+\left(1-y\right)E_{22}$$

The replicator dynamic equation of the QE strategy is

(8) $$f\left(y\right)=dy/dt=y\left(E_{21}-E_{2}\right)=y\left(1-y\right)\left(E_{21}-E_{22}\right)=y\left(1-y\right)\left[x(zI_{q}+R_{q}-C_{q})+z\left(P_{f}+R_{f}\right)+C_{f}-R_{f}\right]$$

The expected payoff of the SG strategy (E_31_), WG strategy (E_32_), and average expected payoff of LGs (E_3_) can be calculated, respectively, by the following:

(9) $$E_{31}=\mathrm{xy}(R_{g}-C_{g})+x\left(1-y\right)\left(P_{f}-C_{g}\right)+\left(1-x\right)y\left(P_{a}-C_{g}\right)+\left(1-x\right)\left(1-y\right)\left(P_{f}-C_{g}\right)$$

(10) $$E_{32}=xyR_{g}-x\left(1-y\right)P_{g}-\left(1-x\right)\left(1-y\right)P_{g}$$

(11) $$E_{3}={\mathrm{zE}}_{21}+\left(1-z\right)E_{22}$$

The replicator dynamic equation of the SG strategy is

(12) $$f\left(z\right)=dz/dt=z\left(E_{31}-E_{3}\right)=z\left(1-z\right)\left[-xyP_{a}+y\left(P_{a}-P_{f}-P_{g}\right)+P_{f}+P_{g}-C_{g}\right]$$

### 2.3. Stability Analysis of the Evolutionary Game Model

When the replicated dynamic equation of the UC strategy, QE strategy, and SG strategy is 0, the system is in equilibrium, that is

(13) $$f\left(x\right)=0,\;f\left(y\right)=0,\;f\left(z\right)=0$$

According to the replicated Dynamic Equation (13), the equilibrium points of the system are $M_{1}=\left(0,\;0,\;0\right)$, $M_{2}=\left(1,0,0\right)$, $M_{3}=\left(0,1,0\right)$, $M_{4}=\left(0,0,1\right)$, $M_{5}=\left(1,1,0\right)$, $M_{6}=\left(1,0,1\right)$, $M_{7}=\left(0,1,1\right)$, $M_{8}=\left(1,1,1\right)$, $M_{9}=\left(x*,y*,z*\right)$; $M_{9}\left(x*,y*,z*\right)$ is the solution to Equation (14)

(14) $$\left(\begin{array}{c} {\mathrm{zS}}_{g}+{\mathrm{zP}}_{a}+yR_{a}+R_{u}-C_{u}+C_{a}-R_{a}=0\\ x(zI_{q}+R_{q}-C_{q})+z\left(P_{f}+R_{f}\right)+C_{f}-R_{f}=0\\ -xyP_{a}+y\left(P_{a}-P_{f}-P_{g}\right)+P_{f}+P_{g}-C_{g}=0 \end{array}\right)$$

The equilibrium point constitutes the boundary of the solution domain $\left\{\left(x*,y*,z*\right)|0<x*<1;0<y*<1;0<z*<1\right\}$, and the surrounding area is the equilibrium solution domain of the three stakeholders. Because the asymptotically stable solution of the multi-agent evolutionary game must be a strict Nash equilibrium, only the equilibrium point M_1_–M_8_ needs to be considered, and the stability of each equilibrium point should be further analyzed.

In this model, the Jacobian matrix is as follows:

(15) $$J=\left(\begin{array}{c} \frac{\partial f\left(x\right)}{\partial x},\\ \frac{\partial f\left(y\right)}{\partial x},\\ \frac{\partial f\left(z\right)}{\partial x}, \end{array}\begin{array}{c} \frac{\partial f\left(x\right)}{\partial y},\\ \frac{\partial f\left(y\right)}{\partial y},\\ \frac{\partial f\left(z\right)}{\partial y}, \end{array}\begin{array}{c} \frac{\partial f\left(x\right)}{\partial z}\\ \frac{\partial f\left(y\right)}{\partial z}\\ \frac{\partial f\left(z\right)}{\partial z} \end{array}\;\right)=\left(\begin{array}{c} a_{11},\\ a_{21},\\ a_{31}, \end{array}\begin{array}{c} a_{12},\\ a_{22},\\ a_{32}, \end{array}\begin{array}{c} a_{13};\\ a_{23};\\ a_{33}; \end{array}\right)$$

$$a_{11}=\left(1-2x\right)\left[{\mathrm{zS}}_{g}+{\mathrm{zP}}_{a}+yR_{a}+R_{u}-C_{u}+C_{a}-R_{a}\right],\;a_{12}=x\left(1-x\right)R_{a},\;a_{13}=x\left(1-x\right)\left(S_{g}+P_{a}\right);\;a_{21}=y\left(1-y\right)\left({\mathrm{zI}}_{q}+R_{q}-C_{q}\right),\;a_{22}=\left(1-2y\right)\left[x\left(zI_{q}+R_{q}-C_{q}\right)+z\left(P_{f}+R_{f}\right)+\left(C_{f}-R_{f}\right)\right];\;\;a_{23}=y\left(1-y\right)\left(xI_{q}+P_{f}+R_{f}\right);\;a_{31}=-z\left(1-z\right)yP_{a},\;a_{32}=z\left(1-z\right)\left(P_{a}-P_{f}-P_{g}\right),\;a_{33}=\left(1-2z\right)\left[-xyP_{a}+y\left(P_{a}-P_{f}-P_{g}\right)+P_{f}+P_{g}-C_{g}\right].$$

When all eigenvalues of the Jacobian matrix are negative, the equilibrium point is an evolutionary stable strategy (ESS). According to the hypothesis of the game system (see Assumption 2.1 of the game model), the positive or negative signs of some eigenvalues can be determined. The eigenvalues of the Jacobian matrix that correspond to each equilibrium point are shown in Table 2. It can be seen in the eigenvalues of the Jacobian matrix that the game system has three different ESS under different conditions. The three ESSs are $M_{1}\left(0,0,0\right)$, $M_{2}\left(1,0,0\right)$, and $M_{5}\left(1,1,0\right)$.

Case 1: It can be seen in Table 2 that two inequalities need to be satisfied simultaneously to achieve Stability Point M_1_ (0,0,0). According to the first inequality, $R_{u}-C_{u}<R_{a}-C_{a}$, when the benefit of the UC strategy is less than the benefit of the AC strategy, the cooperative will choose to produce and provide adulterated raw honey to the beekeeping enterprise. According to the second inequality, $P_{f}+P_{g}<C_{g}$, when the sum of the economic losses and penalties caused by adulterated honey products is less than the supervision cost, the government will choose not to supervise.

Case 2: Three inequalities need to be satisfied simultaneously to achieve Stability Point M_2_ (1,0,0). According to the first inequality, $R_{a}-C_{a}<C_{u}-R_{u}$, when the benefit of the UA strategy is less than the benefit of the UC strategy, the cooperative will choose to produce and provide unadulterated raw honey to the honey product enterprise. According to the second inequality, $R_{q}-C_{q}<R_{f}-C_{f}$, when the benefit of the QE strategy is less than the benefit of the FE strategy, the HE will choose to produce adulterated products. According to the third inequality, $P_{f}+P_{g}<C_{g}$, when supervision costs outweigh the sum of the economic losses and penalties caused by adulterated products, the government will choose not to supervise.

Case 3: There is only one condition that needs to be met to achieve Stability Point M_5_ (1,1,0), that is $R_{f}-C_{f}<R_{q}-C_{q}$. This restriction suggests that the game system will gradually reach (1,1,0) as long as the benefits of qualified honey products outweigh the benefits of adulterated products. 

Through the above stability analysis, the game system has different ESSs under different conditions. M_5_ (1,1,0) is the ideal state among different ESSs. To ensure that the bee product industry can achieve the ideal state, LGs need to ensure through supervision that the benefits of qualified products outweigh the benefits of adulterated products. By using real survey data, we simulated the influence of key factors on the evolution process of the stakeholders under the ideal ESS state.

## 3. Simulation Analysis of Main Influencing Factors

**Figure 3:**
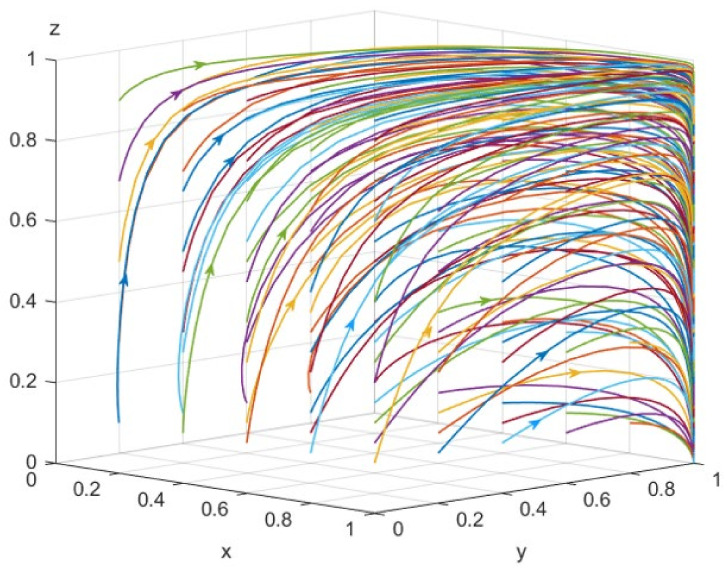
The phase diagram of different stakeholders with initial parameters (Lines of different colors represent evolutionary paths generated from different starting points).

Based on the above model analysis, simulation analysis is used to simulate the dynamic evolution process of the UC strategy, QE strategy, and SG strategy in the game. Parameter values are set in accordance with the literature and market data; we also always refer to the relationship between different parameters in real life. The initial assignment for each parameter is shown in Table 3. The phase diagram with the initial parameters is shown in Figure 3.

In this study, the effects of the five main parameters on the evolutionary process of the game system were evaluated: penalty for adulterated BC (P_a_: from 10 CNY/kg~30 CNY/kg), penalty for adulterated HE (P_f_: from 20 CNY/kg~60 CNY/kg), subsidy to BC (S_g_: from 3 CNY/kg~8 CNY/kg), price of unadulterated raw honey (R_u_: from 16.55 CNY/kg~21.55 CNY/kg) and price of unadulterated bee products (R_q_: from 39.9 CNY/kg~54.9 CNY/kg). These five parameters have more flexible variability and operability in management practice. In the simulation figures, the y-axis represents the probability of a certain strategy. The evolutionary time (i.e., x-axis) stands for the normalized development time after a certain evolution mode begins. It is a normalized time parameter with no unit [40].

### 3.1. Effect of Main Parameters on the Evolutionary Process of the UC Strategy

**Figure 4:**
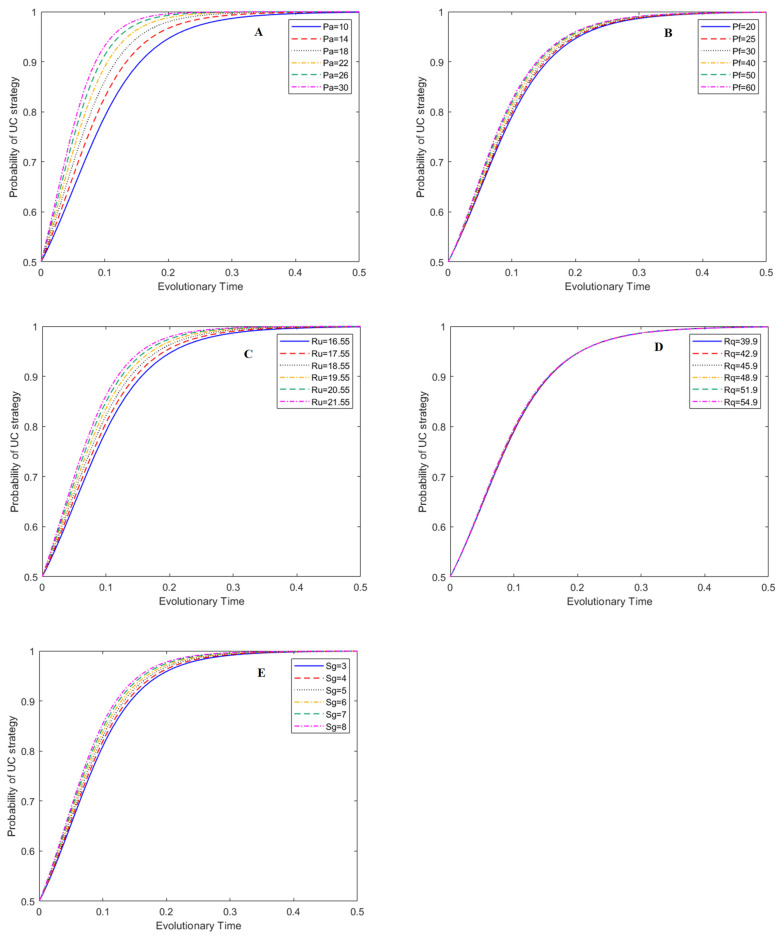
Effects of main parameters on the evolution of UC strategy under (1, 1, 0), subfigures A–E represent the effects of P_a_, P_f_, R_u_, R_q_, and S_g_ on the evolution of UC strategies, respectively.

The effects of P_a_, P_f_, R_u_, R_q_, and S_g_ on the probability of the UC strategy under the ESS of (1, 1, 0) are presented in Figure 4. The results show that P_a_ and R_u_ have obvious effects on the strategy choice of BCs. With the increase in P_a_ and R_u_, the probability of BCs producing unadulterated raw honey increases noticeably (Figure 4A,C). This indicates that LG penalties for adulterated BCs and BCs’ price adjustment of unadulterated raw honey are effective management tools to eliminate the adulteration behavior of BCs.

Figure 4B,E indicate that P_f_ and S_g_ have a smaller degree of influence on the strategy choice of BCs compared to P_a_ and R_u_. With the increase in P_f_ and S_g_, the probability of a BC producing unadulterated honey increases slightly at the same evolutionary time. Figure 4D shows that price adjustment of unadulterated bee products has no impact on the strategy choice of BCs.

### 3.2. Effect of Main Parameters on the Evolutionary Process of the QE Strategy

**Figure 5:**
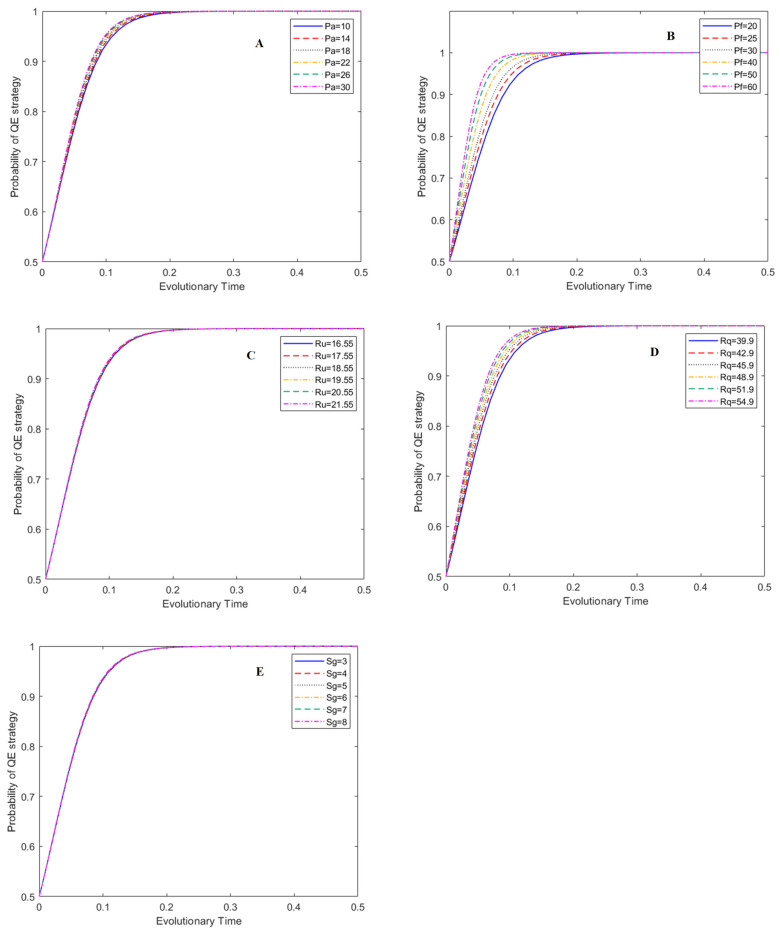
Effects of main parameters on the evolution of QE strategy under (1,1,0), , subfigures A–E represent the effects of P_a_, P_f_, R_u_, R_q_, and S_g_ on the evolution of QE strategies, respectively.

The effects of the five main parameters on the probability of enterprises’ QE strategy under the ESS of (1, 1, 0) are presented in Figure 5. The results show that P_f_ has the most obvious effects on the strategy choice of HE. With the increase in P_f_, the probability of HE producing qualified bee products noticeably increases (Figure 5B), which indicates that LG penalties for adulterated HE is an effective management tool to eliminate the adulteration behavior of HE. Price adjustments of unadulterated honey products also have a positive effect on the probability of the QE strategy. With the increase in R_q_, the probability of HE producing qualified bee products noticeably increases (Figure 5D), although the effect is not as strong as P_f_ at the same evolutionary time. Increasing the price of qualified bee products is an alternative management tool to reduce HE’s adulteration behavior. Figure 5A indicates that P_a_ has a minor influence on BE strategy choice. Figure 5C,E show that R_u_ and S_g_ have no influence on BE strategy choice.

### 3.3. Effect of Main Parameters on the Evolutionary Process of LG’s SG Strategy

**Figure 6:**
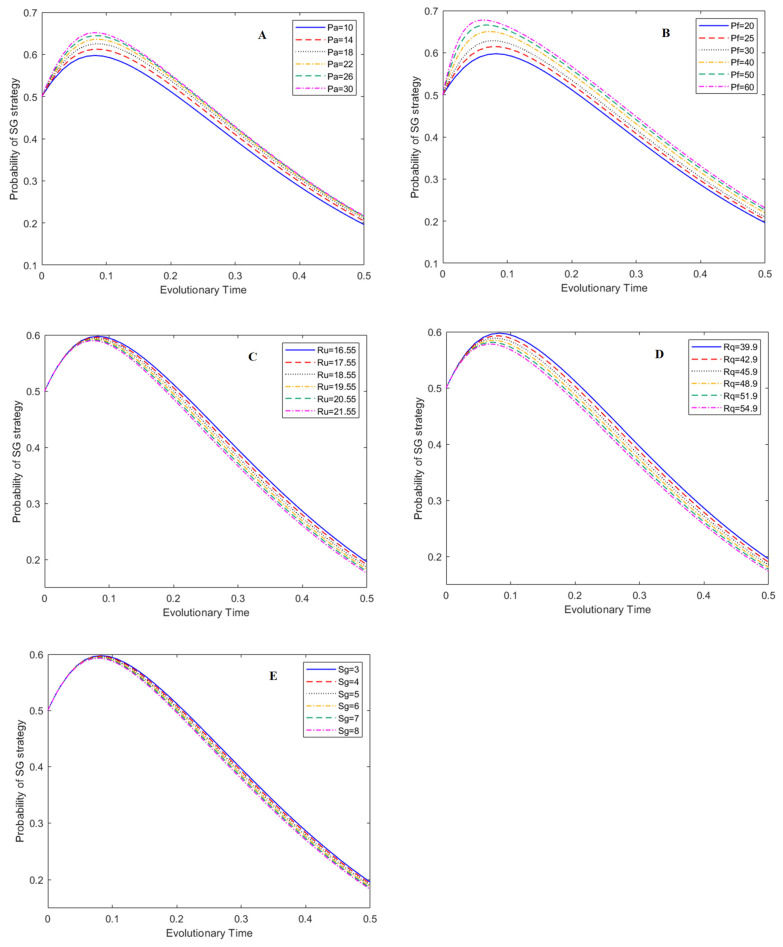
Effects of main parameters on the evolution of SG strategy under (1,1,0), subfigures A–E represent the effects of P_a_, P_f_, R_u_, R_q_, and S_g_ on the evolution of SG strategies, respectively.

The effects of the five main parameters on the probability of the SG strategy under the ESS of (1, 1, 0) are presented in Figure 6. The results show that all five parameters have obvious effects on the strategy choice of LGs. P_f_ and P_a_ have the most obvious influence on the probability of the SG strategy. The probability of LGs to supervise increases with the increase in P_a_ and P_f_ (Figure 6A,B). R_u_ and R_q_ have a medium effect on the probability of the SG strategy at the same evolutionary time. In contrast to the effect of P_a_ and P_f_, the probability of the SG strategy decreases with the increase in R_u_ and R_q_. 

## 4. Discussions and Conclusions 

Apiculture contributes to ecological restoration and poverty eradication in remote regions [41,42]; therefore, the local governments of China have encouraged the development of apiculture by providing free training and financial subsidies for bee farmers. As a result, the quantity of raw honey and honey products has grown rapidly in recent years. Driven by profits, the adulteration of bee products is on the rise [6]. It is urgent that the government supervises the adulteration of honey products. In this paper, a tripartite evolutionary game model consisting of beekeeping cooperatives, enterprises, and governments has been established. The model simulated the behavior of each stakeholder in the process of bee product supervision to provide guidance for honey product supervision. 

The model introduces both subsidy and punishment policies, which is in line with the current situation in China [4,5]. Meanwhile, for the sake of analysis, the model assumes that the implementation of subsidy and punishment strategies for cooperatives is targeted at individual cooperatives rather than individual bee farmers within cooperatives. The paper analyzes the evolution path of beekeeping cooperatives, enterprises, and governments in the game system, aiming to improve government management and speed to end the epidemic of adulterated honey products. Through simulation, this paper illustrates the specific impact of different factors on the evolution of the three parties involved.

(1) A measure that might be used by cooperatives in management practices is to adjust the price of raw honey. As demonstrated by our simulation, if cooperatives increase the price of unadulterated raw honey, they will reach the ESS faster; however, this has no impact on the evolution process of enterprises and governments. By increasing the price of unadulterated raw honey, cooperatives using the UC strategy can gain a bigger profit advantage over cooperatives that use the AC strategy, thus promoting the probability of the UC strategy in cooperatives;
(2) As with cooperatives, if enterprises increase the price of unadulterated bee products, the probability of enterprises using the QE strategy will increase because of the profit advantage it provides compared to the FE strategy;
(3) The behavior of local governments may have an impact on the evolution of the stakeholders in many aspects [32,33,34,36].

Local government’ subsidies to cooperatives have no obvious impact on the evolution process of BCs and BEs. Giving penalties to cooperatives and enterprises that adulterate honey products can effectively increase the proportion of adopting the UC and QE strategies. In the field of new energy and public transport promotion, government subsidies play an important role in promoting the evolution of the system [33,34]. This is because governments use high subsidies to attract stakeholders to use new energy technologies or public transportation [33,34]. However, in the SHP-game model, subsidies from local government usually do not exceed the cost of cooperatives or enterprises; therefore, the impact subsidies have an impact on the value of the overall industry that is too small to affect the actual evolution path of the three parties. Penalties to cooperatives and enterprises that adulterate honey products are generally at least equal to, and sometimes several times greater than, R_a_ or R_f_, which is the same as in the drug supervision game [32]. The simulation shows that the effect of punishment in the SHP-game is the same as that in the drug supervision game, which can effectively change cooperatives’ and enterprises’ behavior.

To sum up, this paper draws important conclusions regarding the SHP-game system.

Generally, local governments’ subsidies to cooperatives have little impact on the evolution path of all stakeholders in the game. LG penalties to BCs and BCs’ price adjustment of unadulterated honey are effective management tools to reduce the adulteration behavior of BCs. LGs’ penalty for adulterated BEs is an effective management tool to reduce the adulteration behavior of BEs.

## Figures and Tables

**Table 1:** Parameter description.

Symbol	Meaning
C_u_	The cost of producing unadulterated raw honey for cooperatives.
R_u_	The price of unadulterated raw honey.
S_g_	Subsidies for cooperatives that produce unadulterated raw honey.
C_a_	The cost of producing adulterated raw honey.
R_a_	The price of adulterated raw honey.
P_a_	Government penalties to cooperatives that produce adulterated raw honey.
C_q_	The cost of producing qualified honey products.
R_q_	The price of qualified honey products.
I_q_	Government incentives to enterprises that produce qualified honey products.
C_f_	The cost of producing adulterated honey products.
R_f_	The price of adulterated honey products.
P_f_	Government penalties to enterprises that produce adulterated honey products.
C_g_	Cost of government supervision.
R_g_	The benefits governments gain from the qualified bee products in the market.
P_g_	Economic losses of governments caused by adulterated products in the market.

**Table 2:** Eigenvalues of Jacobian matrix.

Equilibrium Point	Eigenvalues of Jacobian Matrix			Asymptotic Stability Conditions
	λ_1_	λ_2_	λ_3_	
$M_{1}=\left(0,0,0\right)$	$R_{u}-C_{u}+C_{a}-R_{a}$	$C_{f}-R_{f}$	$P_{f}+P_{g}-C_{g}$	$R_{u}-C_{u}<R_{a}-C_{a}$, $P_{f}+P_{g}<C_{g}$
$M_{2}=\left(1,0,0\right)$	$R_{a}-C_{a}+C_{u}-R_{u}$	$R_{q}-C_{q}+C_{f}-R_{f}$	$P_{f}+P_{g}-C_{g}$	$R_{a}-C_{a}<R_{u}-C_{u}$, $R_{q}-C_{q}<R_{f}-C_{f}$, $P_{f}+P_{g}<C_{g}$
$M_{3}=\left(0,1,0\right)$	$R_{u}-C_{u}+C_{a}$	$R_{f}-C_{f}$	$P_{a}-C_{g}$	Unstable
$M_{4}=\left(0,0,1\right)$	$S_{g}+P_{a}+R_{u}-C_{u}+C_{a}-R_{a}$	$P_{f}+C_{f}$	$C_{g}-P_{f}-P_{g}$	Unstable
$M_{5}=\left(1,1,0\right)$	$C_{u}-R_{u}-C_{a}$	$R_{f}-C_{f}-\left(R_{q}-C_{q}\right)$	$-C_{g}$	$R_{f}-C_{f}<R_{q}-C_{q}$
$M_{6}=\left(1,0,1\right)$	$R_{a}+C_{u}-S_{g}-P_{a}-R_{u}-C_{a}$	$I_{q}+P_{f}+C_{f}+R_{q}-C_{q}$	$C_{g}-P_{f}-P_{g}$	saddle point
$M_{7}=\left(0,1,1\right)$	$S_{g}+P_{a}+R_{u}-C_{u}+C_{a}$	$-P_{f}-C_{f}$	$C_{g}-P_{a}$	saddle point
$M_{8}=\left(1,1,1\right)$	$C_{u}-S_{g}-P_{a}-R_{u}-C_{a}$	$C_{q}-I_{q}-R_{q}-P_{f}-C_{f}$	$C_{g}$	saddle point

**Table 3:** The initial assignment for each parameter.

Parameter	Meaning	Source
C_u_	12.18 CNY/kg	CNY 608.81 per colony [39].
R_u_	16.55 CNY/kg	CNY 827.54 per colony [39].
S_g_	3 CNY/kg	According to the subsidy policy of local governments.
C_a_	4 CNY/kg	Market research.
R_a_	10 CNY/kg	Market research.
P_a_	10 CNY/kg	According to the Product Quality Law in China, 50% to three times the sales of adulterated products.
C_q_	20 CNY/kg	Market research: raw material cost plus labor cost.
R_q_	39.9 CNY/kg	Leading enterprises’ official website factory price.
I_q_	2 CNY/kg	Government incentives come from preferential policies for industry: 1%~10% of product sales.
C_f_	10 CNY/kg	Market research.
R_f_	20 CNY/kg	Market research.
P_f_	20 CNY/kg	According to the Product Quality Law in China, 50% to three times the sales of adulterated products.
P_g_	5 CNY/kg	Inferred from the logical relationship with C_g._
C_g_	5 CNY/kg	Inferred from the logical relationship with S_g_ and I_q._

## Data Availability

Data are available on request.
